# SOX9: a novel janus-faced regulator in immunity and its promise as a therapeutic target

**DOI:** 10.3389/fmolb.2025.1700812

**Published:** 2025-11-10

**Authors:** Qian Shi, Shouyu He

**Affiliations:** 1 Key Laboratory for Translational Medicine, First Affiliated Hospital, The First People’s Hospital of Huzhou, Huzhou University, Huzhou, Zhejiang, China; 2 Department of Spine Surgery, First Affiliated Hospital, The First People’s Hospital of Huzhou, Huzhou University, Huzhou, Zhejiang, China

**Keywords:** SOX9, immunological elements, immune escape, tissue repair, therapeutic

## Abstract

**Background:**

The Sex-determining Region Y-related High-Mobility Group Box 9 (SOX9), a key transcription factor in the SOX family, plays essential roles in various biological processes, particularly in the development of the tumor microenvironment and the repair of inflamed tissues.

**Findings:**

This review elucidates the structure of SOX9 and its relationship with immune components from an immunological perspective, it analyzes the mechanisms by which SOX9 operates in tumor immune escape, inflammatory diseases and tissue repair (such as in osteoarthritis, OA), Furthermore, it introduces a novel immunological perspective by investigating the therapeutic potential and application strategies of SOX9 for treating tumors and inflammatory diseases. and reviews the latest advances in SOX9-related treatments.

**Conclusion:**

SOX9 plays a complex and dual role in immunology, acting as a “double-edged sword”. On one hand, it promotes immune escape by impairing immune cell function, making it a potential therapeutic target in cancer. On the other hand, increased levels of SOX9 help maintain macrophage function, contributing to cartilage formation, tissue regeneration, and repair. Given its significant role in immunobiology, SOX9 represents a promising therapeutic candidate for cancer and immune-related diseases.

## 1 Introduction

The mammalian sex-determining region Y-related high-mobility group box (SOX) family of transcription factors (TFs) was first identified by Gubbay et al., in 1990. These proteins share a defining amino acid sequence homology within an HMG box domain, mirroring that of the mammalian testis-determining factor SRY (Watmough, 2024; Grimm et al., 2020). SOX9, a member of this family, plays an important role in tumor progression, cartilage formation, and stem cell development (Aggarwal et al., 2024; Liu Y. et al., 2022; Yang et al., 2023).

Research has particularly focused on its activating role in tumor biology. SOX9 is frequently overexpressed in various solid malignancies, where its expression levels positively correlate with tumor occurrence and progression (Qian et al., 2024; Borgenvik et al., 2022). Consequently, SOX9 is widely regarded as an oncogene and is significantly implicated in tumor chemoresistance and malignant potential (Panda et al., 2021). Beyond its established roles in chondrogenesis and tumorigenesis, SOX9 is expressed in embryonic liver and pancreatic progenitor cells, serving as a marker for hepatic and pancreatic stem/progenitor cell populations (Kawaguchi, 2013). Furthermore, accumulating evidence highlights significant connections between SOX9 and immune system regulation (Luo et al., 2022).

SOX9 exhibits context-dependent dual functions—acting as both an activator and a repressor—across diverse immune cell types, thereby contributing to the regulation of numerous biological processes (Liu Y. et al., 2023; Chen et al., 2021; Michelatti et al., 2024). Immune responses are broadly categorized as innate or adaptive, distinguished by their specificity and kinetics. Innate immunity provides rapid, non-specific defense mediated by components such as acute-phase proteins, neutrophils, monocytes, macrophages, complement, and cytokines (Demaria et al., 2019). In contrast, adaptive immunity, a hallmark of higher vertebrates, is characterized by its specificity and memory, involving antigen-specific responses orchestrated by T and B lymphocytes; however, these responses require days to weeks to fully develop (Mantovani and Garlanda, 2023; Parkin and Cohen, 2001). Leveraging the exquisite specificity of the adaptive immune system for therapeutic purposes, particularly against cancer, has driven significant advancements since the early 20th century. Key immunotherapeutic strategies include adoptive cell transfer (ACT) (Met et al., 2019), chimeric antigen receptor T-cell (CAR-T) therapy (Sterner and Sterner, 2021). Monoclonal antibodies targeting immune checkpoint pathways, such as cytotoxic T-lymphocyte-associated protein 4 (CTLA-4) and programmed cell death protein 1 (PD-1) (Ribas and Wolchok, 2018; Rowshanravan et al., 2018; Yi et al., 2022; Adusumilli et al., 2021). These approaches have been used successfully against multiple cancers, including both hematological and solid tumors.

## 2 Structural and immunological characteristics of SOX9

**FIGURE 1:**
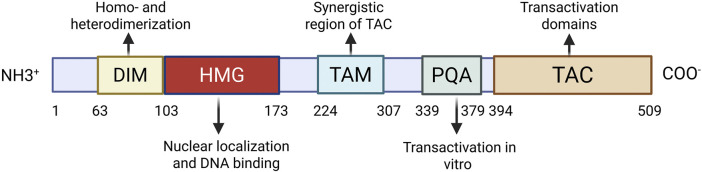
An illustration of the human SOX9 protein. Ahead of the HMG box lies the dimerization domain (DIM). There are two transcriptional activation domains at the C-terminus (TAC) and in the middle (TAM). For transcriptional activation, the proline, glutamine, and alanine (PQA)-rich domain is necessary. Each domain’s primary purposes are listed.

The High Mobility Group (HMG) box, an evolutionarily conserved DNA-binding motif, is the defining feature of the SOX family. SOX9, a member of this SRY-related HMG box protein family, encodes a 509 amino acid polypeptide crucial for cartilage development, sex determination, and embryogenesis (Underwood et al., 2023). This protein contains several functional domains organized from N- to C-terminus: a dimerization domain (DIM), the HMG box domain, two transcriptional activation domains–one central transcriptional activation domain (TAM) and one at the C-terminus (TAC) – and a proline/glutamine/alanine (PQA)-rich domain (Geraldo et al., 2016) (Figure 1). The HMG and transcriptional activation domains are primarily responsible for SOX9’s core functions. The HMG domain serves dual roles: it directs nuclear localization via embedded (Argentaro et al., 2003) localization (NLS) and export (NES) signals, enabling nucleocytoplasmic shuttling, and facilitates DNA binding (Argentaro et al., 2003; Tsuchiya et al., 2011). The C-terminal transcriptional activation domain (TAC) interacts with diverse cofactors, such as Tip60, to enhance SOX9’s transcriptional activity (Haseeb and Lefebvre, 2019). TAC is also essential for β-catenin inhibition during chondrocyte differentiation (Akiyama et al., 2004). The TAM functions synergistically with TAC to augment the transcriptional potential of SOX9 (Haseeb and Lefebvre, 2019).

SOX9 (SRY-box 9) plays a significant role in immune cell development, participating in the differentiation and regulation of diverse immune lineages. Regarding T cell development, a SOX9 can cooperate with c-Maf to activates Rorc and key Tγδ17 effector genes (Il17a and Blk), which modulates the lineage commitment of early thymic progenitors, potentially influencing the balance between αβ T cell and γδ T cell differentiation (Parker and Ciofani, 2020; Huang et al., 2002). Within the B cell lineage, SOX9 does not have a known significant role in normal B cell development. However, SOX9 is overexpressed in certain types of B-cell lymphomas, like Diffused Large B-cell Lymphoma (DLBCL), where it acts as an oncogene by promoting cell proliferation, inhibiting apoptosis, and contributing to the cancer’s progression (Shen et al., 2022). Given its critical role in orchestrating immune cell differentiation, SOX9 holds significant therapeutic potential for diseases caused by immune system dysregulation.

## 3 SOX9 in cancer

Cancer is a complex disease, and dysregulation of transcription factors is common in cancer pathogenesis. The SOX family is an important family of stem cell transcription factors, among which SOX9 is highly expressed in a variety of cancers, such as liver cancer (Ma X. L. et al., 2020), lung cancer (Olsen et al., 2021), breast cancer (Christin et al., 2020), Gastric cancer (Chen et al., 2023), etc. It is a downstream target of several embryonic signaling pathways and has a close relationship with vascularization (Faleeva et al., 2024), drug resistance (Tripathi et al., 2022), tumor proliferation (Liu Z. et al., 2022), metastasis (Qi and Li, 2020), and apoptosis (Shen et al., 2022). Additionally, SOX9 is strongly linked to tumor cells' poor prognosis (Qian et al., 2024). Transcriptional regulation and post-transcriptional regulation are the two primary categories of regulatory mechanisms. Changes in epigenetic alterations like methylation and acetylation are included in transcriptional regulation (Sun et al., 2023; Xie et al., 2021), whereas biological activities mediated by miRNA and lncRNA are primarily included in post-transcriptional regulation (Ashrafizadeh et al., 2021; He et al., 2021).

### 3.1 Relationship between SOX9 and tumor immune cell infiltration

**FIGURE 2:**
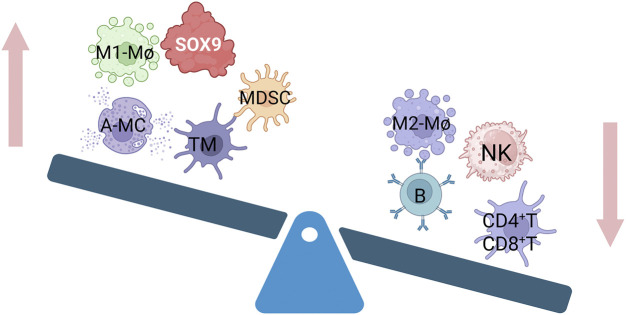
Relationship between different immune cell infiltration and SOX9 overexpression. Memory T cells (TM), polymorphonuclear myeloid-derived suppressor cells (MDSCs), activated mast cells (A-MC), and M1 macrophage (M1-Mø) infiltration are all favorably connected with overexpressed SOX9. It has a negative correlation with M2 macrophages(M2-Mø), B cells, NK cells, and CD4^+^T and CD8^+^T infiltration.

Extensive bioinformatics analyses indicate a strong association between SOX9 expression and immune cell infiltration within tissues. For instance, by integrating whole exome and RNA sequencing data from The Cancer Genome Atlas, Chong Wang et al. identified SOX9 as a characteristic gene for early and late diagnosis of colorectal cancer (CRC). SOX9 expression negatively correlated with infiltration levels of B cells, resting mast cells, resting T cells, monocytes, plasma cells, and eosinophils, but positively correlated with neutrophils, macrophages, activated mast cells, and naive/activated T cells (Wang et al., 2021). Similarly, Hua Zhong et al. demonstrated that SOX9 overexpression negatively correlates with genes associated with the function of CD8^+^ T cells, NK cells, and M1 macrophages, while showing a positive correlation with memory CD4^+^ T cells (Zhong et al., 2023; Du et al., 2023; Zhao Y. et al., 2024) (Figure 2). In single-cell RNA sequencing and spatial transcriptomics analyses of prostate cancer (PCa) patients and healthy controls, effector immune cells were observed a shift in the immune landscape, such as CD8^+^CXCR6^+^ T cells and activated neutrophils, are decreased, while immunosuppressive cells, including Tregs, M2 macrophages (TAM Macro-2), and anergic neutrophils, are increased. This imbalance ultimately creates an “immune desert” microenvironment that promotes tumor immune escape. At the same time, long-term androgen deprivation therapy (ADT) may indirectly weaken the anti-tumor immune response by enriching a subpopulation of club cells characterized by high SOX9 and low AR (SOX9high ARlow) (Bian et al., 2024). Chengqian Zhong et al. further classified CRC into three molecular subtypes (CS1, CS2, CS3) based on transcriptomic, DNA methylation, and driver mutation data. Notably, the CS3 subtype exhibited higher immune cell infiltration and significant SOX9 upregulation (Zhong et al., 2022). The above research highlights SOX9’s pivotal role in modulating immune infiltration, providing support for targeting SOX9 to improve patient prognosis. Current research on SOX9’s regulatory mechanisms primarily focuses on its impact on inflammatory diseases (e.g., tumors (Jana et al., 2020), kidney damage (Chen et al., 2022), pneumonia (Zhu et al., 2022)), and the effects on the functions of T cells (Ramakrishnan et al., 2023), NK cells (Zhong et al., 2023) and macrophages (Feng D. et al., 2023), However, its regulatory effects on B cells and mast cells remain less explored.

### 3.2 Mechanism of SOX9 involvement in tumor immune escape

**FIGURE 3:**
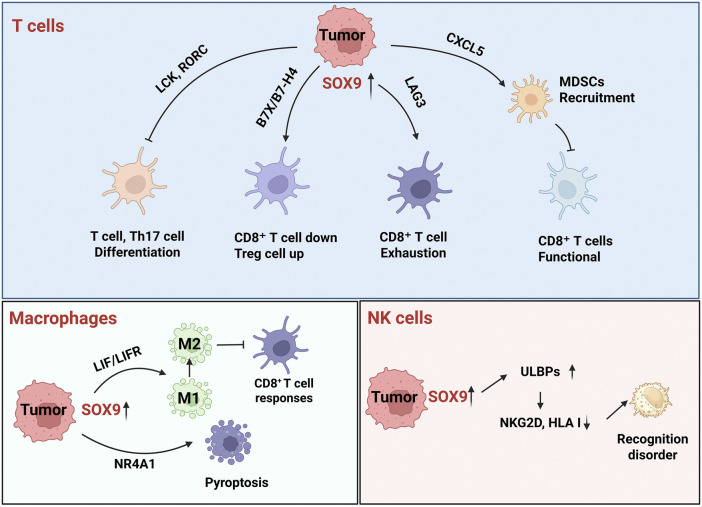
Mechanism of SOX9 regulating tumor immune escape. SOX9 regulates biological processes such as T cell development, infiltration, and exhaustion through various pathways, promotes macrophage polarization and pyroptosis, and escapes NK cell immune surveillance. LCK, LCK proto-oncogene; RORC: RAR related orphan receptor C; B7_X_: Immune checkpoint; LAG3: Lymphocyte activating 3; CXCL5: C-X-C motif chemokine ligand 5; LIF: Leukaemia inhibitory factor; NR4A1: Nuclear receptor subfamily 4 group A; ULBPS: UL16-binding proteins; NKG2D: Killer cell lectin like receptor K1 (KLRK1); HLA: Major histocompatibility complex.

SOX9 is a master regulator of tumor immune evasion. It is a highly promising therapeutic target. Its ability to suppress anti-tumor immunity through multiple, synergistic pathways, makes it a central node in the network of cancer immune resistance (Table 1). According to the mechanism by which SOX9 regulates various immune components, its mechanism of involvement in tumor immune escape is as follows (Figure 3).

**TABLE 1:** Function of SOX9 in immune-related diseases.

Immune-related diseases		SOX9 targets	Immune effect	Refs
Tumor	Gastric cancer	LIF/LIFR	Promoting the differentiation of M2 macrophages in the tumor microenvironment	Fan et al. (2023)
	Pancreatic cancer	CXCL5/CXCR2	Recruitment of polymorphonuclear myeloid-derived suppressor cells (MDSCs), which will speed up the growth of the tumor and block T lymphocyte function	Chen et al. (2021)
	Breast cancer	B7x/B7-H4	Reduces CD8 + T cells and increases the infiltration of regulatory T cells (Treg cells)	Liu Y. et al. (2023)
Osteoarthritis(OA)		NF - κB	promotes macrophage polarization from M1 to M2	Tian et al. (2024)
Pulmonary fibrosis		IL-4Ra	Released by regulatory T cells (Tregs) and interstitial macrophages	Cai et al. (2024)
Dental pulp		TNF-α	influences the extracellular matrix composition, cytokine production	Luo et al. (2018)

#### 3.2.1 SOX9 and T cells

T cells, categorized as T helper (Th, CD4^+^) or cytotoxic T (Tc, CD8^+^) cells, are central to adaptive immunity. CD4^+^ Th cells coordinate immune responses by recognizing antigens and secreting cytokines (e.g., interferons) to activate macrophages. CD8^+^ Tc cells directly kill antigen-bearing target cells by releasing perforin and granzymes, thereby inducing apoptosis (Galli et al., 2023; Gonzalez-Rodriguez et al., 2022).

Elevated SOX9 expression in tumors correlates with reduced T cell infiltration, facilitating tumor immune escape and progression (Bian et al., 2024).

*In vivo* studies have shown that in thymoma patients with high SOX9 expression, the infiltration of CD4^+^ T cells and CD8^+^ T cells were significantly reduced, while macrophage infiltration was increased. Notably, M2 macrophages accounted for 10% of the total infiltrating immune cells in the high SOX9 expression group. High SOX9 expression is negatively correlated with the expression of LCK and RORC (genes involved in the development function, and differentiation of T cells and Th17 cells), suggesting *In vitro* and animal model studies support that SOX9 significantly inhibits the development, differentiation, and tumor-killing function of T cells and Th17 cells (Yuan et al., 2021). In SOX9-overexpressing lung adenocarcinoma, tumor-infiltrating T cells, particularly CD8^+^ T cells, exhibited elevated levels of the exhaustion marker LAG3, indicating that SOX9 promotes CD8^+^ T cell dysfunction and exhaustion (Zhong et al., 2023). In pancreatic ductal adenocarcinoma, the SOX9–CXCL5 axis promotes the recruitment of polymorphonuclear myeloid-derived suppressor cells (PMN-MDSCs), which accelerate tumor progression and suppress T lymphocyte function (Luo et al., 2022). Conversely, SOX9 knockdown enhances immune cell infiltration. Co-culture of SOX9-knockout tumor cells with CD45^+^ T cells or peripheral blood mononuclear cells (PBMCs) significantly increased CD8^+^ T cell responses, an effect independent of NK cells (Fan et al., 2023). Additionally, it has been shown that SOX9 knockdown can greatly increase the quantity and cytotoxic capacity of CD8^+^ T cells. More significantly, it can restore the immune function of exhausted CD8^+^ T cells. These cells are found in the ascites of patients with gastric cancer peritoneal metastases, highlighting its clinical relevance for treating this condition. These findings collectively position SOX9 as a potent driver of immune evasion. It employs multiple mechanisms, both direct and indirect, to suppress T cell function. This makes SOX9 inhibition a compelling strategy to improve cancer immunotherapy.

#### 3.2.2 SOX9 and NK cells

Natural killer (NK) cells provide frontline defense against cancer and infected cells through immune surveillance, directly recognizing and eliminating abnormal cells without prior sensitization (Terren et al., 2019; Liu et al., 2021). They can directly recognize and kill cancer cells without requiring activation or sensitization by other immune cells. Similar to CD8^+^ T cells, NK cells secrete perforin onto the surface of target cells, increasing membrane permeability, and release granzymes that induce apoptosis (Shimasaki et al., 2020). Normal host cells express MHC class I molecules, which bind to inhibitory receptors on NK cells and suppress the killing pathway. In contrast, tumor cells and virus-infected cells (especially those from the herpesvirus family) often exhibit reduced levels of MHC class I proteins, making them more susceptible to NK cell-mediated attack (Neo et al., 2022; Saunders et al., 2015).

High expression of SOX9 inhibits the infiltration of NK cells into tumors, and several studies have explored its regulatory mechanisms. For instance, in breast cancer models, NK cells were shown to restrict the growth of latency-competent cancer (LCC) cells when injected into immunocompetent athymic animals. However, SOX9 overexpression enabled these tumor cells to evade immune surveillance, possibly by upregulating inhibitory ligands such as UL16-binding proteins (ULBPs), Which a novel MHC class I-related molecules, bind to CMV glycoprotein UL16 and stimulate NK cytotoxicity through the NKG2D receptor (Malladi et al., 2016), This suggests that SOX9 may play a crucial role in helping tumor cells escape NK cell-mediated killing. In addition, studies on disseminated tumor cells (DTCs) have shown that retinoic acid (RA) signaling can induce tumor dormancy by modulating SOX9 expression, and NK cell receptors (such as NKG2D ligands and HLA class I genes) also significantly decreased (Michelatti et al., 2024). SOX9 emerges as a critical orchestrator of resistance to innate immune surveillance by NK cells. Its ability to simultaneously reduce NK cell recruitment to the tumor and, more importantly, engineer the tumor cell surface to evade recognition—by both suppressing “eat-me” signals (activating ligands) and potentially enhancing “don’t-eat-me” signals (inhibitory ligands)—represents a powerful and multifaceted strategy for immune escape.

#### 3.2.3 SOX9 and macrophages

Macrophages serve as a bridge between innate and adaptive immunity. They can engulf and digest foreign pathogens and dead cells, present antigens to T cells to activate specific immune responses, and release inflammatory factors (such as tumor necrosis factor-α and interleukin-1β) to attract other immune cells to sites of infection or injury. They also release growth factors to promote cell proliferation and tissue regeneration (Wynn and Vannella, 2016; Guilliams and Scott, 2022). The expression of SOX9 is positively correlated with macrophage infiltration. Studies by Yibo Fan et al. have shown that knocking out SOX9 can inhibit the expression of leukemia inhibitory factor (LIF), thereby promoting the polarization of M2 macrophages in the tumor microenvironment. This reduces the production of CCL2 and IL-10 by macrophages and facilitates tumor immune escape and metastasis (Fan et al., 2023). Thus, SOX9 modulates macrophage polarization, pyroptosis, and activity, playing significant roles in tumor immune evasion.

#### 3.2.4 SOX9 and immune checkpoints

Immune checkpoint molecules regulate the activity of immune cells, maintain immune system balance, and prevent excessive immune activation from attacking the body’s own tissues, thereby avoiding the occurrence of autoimmune diseases (Postow et al., 2018; Topalian et al., 2016). SOX9 expression is closely associated with the expression of immune checkpoints across various cancer types. Gene co-expression analysis was performed in hepatocellular carcinoma (HCC). It revealed that SOX9 is significantly positively correlated with 47 known immune checkpoint genes. These include well-established clinical targets such as PD-1, PD-L1, and CTLA4 (Luo et al., 2022). Research by S. Liu et al. also demonstrates that SOX9 expression is closely linked to other tumor immune checkpoints, such as CD27 (Liu S. et al., 2023). SOX9-mediated immune suppression is essential for the progression from *in situ* tumors to invasive cancer. The specific mechanism, as elucidated in a 2023 study by CC et al., shows that SOX9 is activated via STAT3 and directly regulates the expression of the immune checkpoint molecule B7x/B7-H4. This leads to a reduction in CD8^+^ T cells and an increase in regulatory T cell (Treg) infiltration, thereby re-establishing an immunosuppressive microenvironment and accelerating tumor development (Liu Y. et al., 2023). B7x not only supports mammary gland regeneration in immunocompetent mice but can also be targeted in advanced tumors to inhibit their growth and overcome resistance to anti-PD-1 immunotherapy (Pulanco et al., 2023; Li et al., 2018). Collectively, studies across mouse models, cell lines, and patient samples define SOX9 as a regulator of immune checkpoints. They also reveal a promising therapeutic strategy: targeting the SOX9-B7-H4 axis. This potential is notably high in basal-like breast cancer.

#### 3.2.5 SOX9 and chemokines

Chemokines constitute a specialized subfamily of cytokines that play a key role in leukocyte migration (Mughees et al., 2022). They promote the recruitment of innate immune cells to sites of infection, regulate adaptive immune responses—including immune activation and memory—and modulate effector cell functions (Vilgelm and Richmond, 2019; Jiang et al., 2020). Several studies have reported SOX9-mediated regulation of chemokines. For example, in SOX9-overexpressing tumors, the levels of the chemokine receptor CXCR3 and its ligands (CXCL9 and CXCL10), as well as CXCR6 and its ligand CXCL16, were significantly reduced. Additionally, expression of Flt3, an important gene for dendritic cell development, was markedly decreased. These changes may contribute to the inhibition of tumor-infiltrating dendritic cells by SOX9, thereby suppressing CD8^+^ T cells and NK cells (Zhong et al., 2023). Other studies have shown that cancer cells are the main producers of CXCL5, and that its expression is directly regulated by SOX9 (Ren et al., 2022). CXCL5 is a potent chemokine that recruits polymorphonuclear myeloid-derived suppressor cells (PMN-MDSCs), which inhibit the function of T and B lymphocytes, and significantly impair lymphocyte homing and the activation of multiple key immune signaling proteins (Chen et al., 2021; Yang et al., 2024). Thus, SOX9 promotes the recruitment of immunosuppressive MDSCs through chemokine regulation—particularly via CXCL5—thereby inhibiting effector lymphocytes and facilitating tumor immune escape.

## 4 SOX9 in immune-related diseases

### 4.1 Role of SOX9 in osteoarthritis (OA)

Osteoarthritis (OA) is a common disease that affects more than 500 million people worldwide and currently lacks an effective cure. Traditional treatments such as physical therapy, painkillers, and joint replacement surgery have certain limitations (Martel-Pelletier et al., 2016). Early studies have shown that SOX9 is a major transcription factor in cartilage development and endochondral ossification (Bell et al., 1997), and is essential for cartilage extracellular matrix (ECM) gene expression and chondrocyte differentiation (Lefebvre et al., 2019). SOX9 can promote gene transcription of cartilage markers type II and type IX collagen and proteoglycan aggrecan, and inhibit the expression of aggrecanase and matrix metalloproteinases, thereby maintaining the phenotype and normal function of articular chondrocytes (Imagawa et al., 2014; Zhang et al., 2006; McKillop et al., 2013; van Beuningen et al., 2014). The latest studies related to immune regulation have shown that in the pathological process of OA. In chondrocytes, NF-κB regulation of SOX9 is promotive. NF-κB, after entering the nucleus, may exert its regulatory effects by binding to the SOX9 promoter to form a SOX9-p65-NF-κB complex. NF-κB have a function of promotes macrophage polarization from M1 (proinflammatory) and M2 (anti-inflammatory) (Fang et al., 2024), thereby affecting the physiological state of cartilage (Tian et al., 2024). However, under stimulation by inflammatory factors (such as IL-1 and TNF-α), SOX9 expression is restricted, which increases the severity of arthritis.

### 4.2 Role of SOX9 in tissues repair

The involvement of immune cells in tissue repair is intricately associated with the transcription factor SOX9, which has been shown to facilitate tissue repair and functional recovery (Liu J. A. et al., 2023). In the context of immune-mediated repair, SOX9 emerges as a pivotal intrinsic molecular regulator in the renal epithelial regeneration response following acute kidney injury (AKI). Notably, 24 h post-ischemia-reperfusion injury (IRI), zinc finger protein 24 (ZFP24) was found bound to a specific site in both murine and human SOX9 promoters (Kim et al., 2023). Kidney repair after AKI depends on the regeneration of epithelial cells and macrophages. Specifically, activated SOX9+ epithelial cells secrete cytokines (C3) and growth factors (S100A9), which modulate macrophage activity to jointly facilitate renal repair (Kumar, 2018; Guo et al., 2023; Nie et al., 2023). Cai et al. (2023) have demonstrated that microenvironments associated with inflammation and aging can contribute to the persistent activation of pulmonary fibrosis. Regulatory T cells (Tregs) and interstitial macrophages serve as significant sources of interleukin-4 (IL-4). The IL-4 produced by these immune cells can stimulate the expression of the transcription factor SOX9 in type II alveolar epithelial cells (ATIIs), thereby modulates macrophage activity and reprograms lung epithelial cells into a progenitor-like state. This new state holds dual differentiation potential—for both airway and alveolar lineages. Conversely, in a bleomycin-induced model of pulmonary fibrosis, diminished levels of SOX9 expression and the lack of IL-4 signaling through IL-4Ra in macrophages exacerbate the condition. This finding suggests that aberrant SOX9 expression may indirectly impair the functionality of immune cells involved in the differentiation and repair of alveolar cells (Cai et al., 2024).

### 4.3 Role of SOX9 in other inflammatory diseases

**FIGURE 4:**
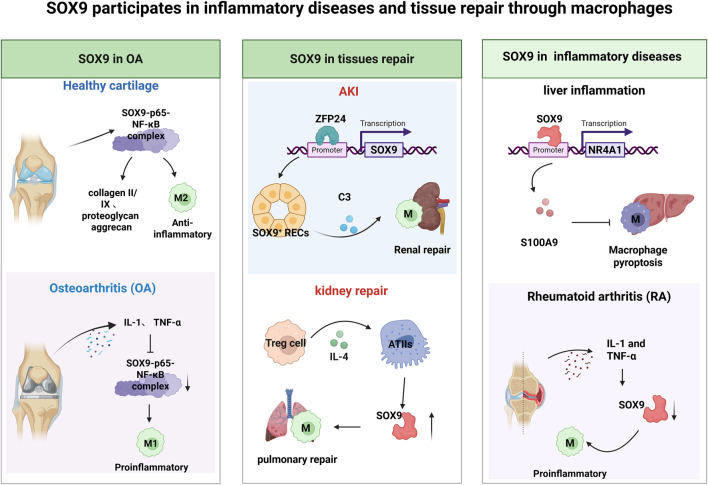
SOX9 in immune-related diseases SOX9 plays a beneficial role in inflammatory diseases and tissue repair. In healthy cartilage, SOX9 interacts with p65 and NF-κB to form a complex, promotes the production of collagen and the proteoglycan aggrecan, and facilitates the switch in macrophage polarity from the pro-inflammatory M1 phenotype to the anti-inflammatory M2 phenotype. However, in osteoarthritis (OA), these regulatory processes are disrupted, and M1 macrophages dominate, promoting a pro-inflammatory environment. Zinc finger protein 24 (ZFP24) has been found to bind to the SOX9 promoter. Activated SOX9^+^ renal epithelial cells (SOX9^+^ RECs) promote kidney repair by secreting the cytokine C3, which influences macrophage activity and collectively contributes to the renal repair process. Regulatory T cells (Tregs) are a major source of interleukin-4 (IL-4). IL-4 can stimulate the expression of the transcription factor SOX9 in type II alveolar epithelial cells (ATIIs), thereby modulating macrophage activity and promoting the reprogramming of lung epithelial cells. SOX9 acts as a transcriptional coactivator of nuclear receptor subfamily 4 group A member 1 (NR4A1), interacting with it to regulate the expression of the downstream gene S100A9. This interaction alleviates macrophage pyroptosis but exacerbates liver inflammation. In rheumatoid arthritis (RA), IL-1 and TNF-α downregulate SOX9 expression, which impairs macrophage function.

In addition to its involvement in tumor immune evasion and tissue repair mechanisms, SOX9 is implicated in various immune-related disorders. For instance, in the context of dental pulp inflammation and immune response, the expression of SOX9 is significantly suppressed by tumor necrosis factor α (TNF-α), which subsequently influences the extracellular matrix composition, cytokine production, and overall immune response within the dental pulp (Luo et al., 2018). Other studies have indicated that in livers lacking Caspase 6, activation of SOX9 in macrophages exacerbates liver inflammation. The specific mechanism involves SOX9 acting as a transcriptional coactivator of nuclear receptor subfamily 4 group A member 1 (NR4A1), interacting with it to regulate the expression of the downstream gene S100A9. This alleviates macrophage pyroptosis and aggravates liver inflammation (Sheng et al., 2023). Furthermore, alterations in SOX9 expression have been observed in joint synovial cells in the context of rheumatoid arthritis (RA), where abnormal proliferation and inflammatory responses of these cells are characteristic features of the disease. It is posited that SOX9 may contribute to the pathogenesis of RA by modulating the biological behaviors of synovial cells, including their proliferation, extracellular matrix secretion, and interactions with immune cells (Li G. et al., 2024). in the Intestinal tissues of patients with inflammatory bowel disease (IBD), including Crohn’s disease (Kobayashi et al., 2024; Zhuang et al., 2022), and as well as in conditions such as Sjögren’s syndrome abnormalities in SOX9 expression are frequently noted (Xiang et al., 2023). Additionally, analyses at both the single-cell level (using patch-based single-cell transcriptomics) and the tissue level (using spatial transcriptomics) revealed that SOX9 may participate in brain injury repair by regulating synaptic homeostasis and immune responses. SOX9 deficiency results in reduced expression of microglial activation markers, such as Cxcl10 and Serpina3n. This suggests that aberrant SOX9 expression may be linked to neurodegenerative diseases or neuroinflammation, thereby providing a theoretical foundation for astrocyte-targeted therapeutic strategies (Natarajan et al., 2025). These observations suggest that, in contrast to its role in tumor immune escape, SOX9 plays a positive role in the pathophysiology of autoimmune and inflammatory diseases by maintaining macrophage function, which warrants further investigation (Figure 4).

## 5 Therapeutic implications and strategies of SOX9

For in tumor, numerous studies have demonstrated that SOX9 influences tumor resistance via multiple signaling pathways, including PI3K/AKT (Wang L. et al., 2020), Wnt/β-catenin (Ramakrishnan et al., 2023; Feng Q. et al., 2023), and TGFβ/Smad (Ma et al., 2023). This modulation plays a critical role in the persistence of various cancers, including gastric, liver, and breast cancer, as well as in the efficacy of treatments such as sorafenib and tamoxifen (Wang M. et al., 2020; Xue et al., 2019). Given its critical role in tumor dormancy and NK cell evasion, SOX9 is a key strategic target. Disrupting its signaling pathways may effectively awaken dormant tumors and enhance immune attack. Furthermore, in the context of resistance to immunotherapy, the expression of PD-L1 has been linked to immune evasion. Given that SOX9 indirectly influences PD-L1 expression through its impact on cell phenotype, it has the potential to reduce the resistance of melanoma cells to immunotherapeutic strategies, including immune checkpoint inhibitor therapy (Subhadarshini et al., 2023). This strategy offers a theoretical foundation for the advancement of innovative tumor immunotherapy approaches.

SOX9 provides a therapeutic benefit in inflammatory diseases and tissue repair, notably by mitigating osteoarthritis and alleviating acute kidney injury. One key mechanism is the maintenance of macrophage activity. This strategy can be implemented by leveraging stem cell secretions or nanomaterial-based delivery systems. Moreover, its potential in treating immune-related diseases warrants further attention, as elaborated in the following sections (Table 2).

**TABLE 2:** Treatment technologies construction based on SOX9.

Treatment technologies	SOX9 up or down	System construction method	Diseases	Ref
siRNA or shRNA	Down	Depleted by small interfering RNA	Colorectal cancer, Hepatocellular carcinoma, Non-small cell lung cancer, Gastric adenocarcinoma, Triple-Negative Breast Cancer	Ma X. L. et al. (2020), Fan et al. (2023), Zhou et al. (2020), Yan et al. (2024), Ye et al. (2024)
	Down	Depleted by small interfering RNA	Thyroid Eye Disease	Zhou et al. (2024)
CRISPR	Down	SOX9-CRISPR/Cas9	lung adenocarcinoma (LUAD), Hepatocellular Carcinoma-Cholangiocarcinoma	Zhong et al. (2023), Park et al. (2024)
	Up	SOX9-CRISPR-dCas9 technology	Osteonecrosis of the femoral head (ONFH)	Meng and Zhu (2023)
	Up	SOX9-CRISPR-dCas9 technology	Osteoarthritis (OA)	Zhao L. et al. (2024)
	Up	SOX9-CRISPR-dCas9 technology	Acampomelic campomelic dysplasia (ACD)	Mochizuki et al. (2018)
Nanoparticle	Down	cRGDfK peptide-modificed LNPs (R-LNPs) composed of DLin-MC3-DMA, DMG-PEG, DSPC, DSPE-PEG-cRGDfK, and cholesterol for the targeted delivery of SOX9 siRNA (siSOX9)	Colorectal Cancer	Zhang et al. (2025)
	Up	Poly-lactic-co-glycolic acid (PLGA) nanoparticle plasmid delivery system	Ischemic brain damage	Shin et al. (2024)
	Up	Dexamethasone-conjugated polyethylenimine (DEXPEI) complexed with minicircle plasmid (MC) harboring SOX duo (SOX-9, -6)	Osteoarthritis (OA)	Jeong et al. (2020)
Extracellular vesicle	Up	EVs derived from human placenta-derived MSCs (hP-MSCs) to a Sox9-CreERT2	Acute kidney injury (AKI)	Zhang et al. (2020)
Peptide vaccine	None	The immunodominant regions from the SOX9 protein were fused using appropriate linkers (EAAAK, KK, AAY and GPGPG) and adjuvant (50S ribosomal protein L7/L12) to enhance the vaccine’s immunogenicity	Triple-negative breast cancer	Rajendran Krishnamoorthy and Karuppasamy (2023)
Cells injection	Up	CuO@MSN/Sox9/Bmp7 (CSB NPs) -engineered MSCs	Osteoarthritis (OA)	Wu et al. (2025)
Targeted protein degradation	Up	Fatty acid oxidation (FAO) promotes SOX9 ubiquitination-mediated degradation	Osteoarthritis (OA)	Mei et al. (2025)

### 5.1 Directly targeting SOX9 by CRISPR

CRISPR is a gene-editing system derived from a bacterial immune mechanism. It utilizes a guide RNA and the Cas9 protein to make precise cuts in DNA, allowing scientists to modify genomes for various applications. A SOX9 CRISPR/Cas9 system employs this technology to edit the SOX9 gene. This process involves designing a single-guide RNA (sgRNA) that recognizes a specific sequence within the SOX9 gene, directing the Cas9 enzyme to cleave the DNA at that site.

As noted previously, SOX9 can influence immune checkpoints such as LIF, PD-1, and B7x. Using CRISPR/Cas9 to target these checkpoints, along with M2 macrophages, has been shown to enhance innate immunity and T cell responses. This leads to a significant reduction in tumor metastasis and changes in the tumor microenvironment (Liu Y. et al., 2023; Fan et al., 2023). Recent advances have expanded the CRISPR toolkit to include SOX9 CRISPR activation (CRISPRa) with a catalytically deactivated Cas9 (dCas9). This approach uses a modified CRISPR system to upregulate the expression of SOX9, a master regulator of chondrogenesis (cartilage formation). By activating SOX9, CRISPRa improves the capacity of cells—such as mesenchymal stem cells (MSCs)—to differentiate into cartilage-producing cells. Engineered MSCs with enhanced chondrogenic potential exhibit reduced macrophage infiltration and decreased IL-1β production. This strategy has shown promising results in preclinical mouse models of osteoarthritis (OA), promoting cartilage repair, mitigating cartilage destruction, and reducing pain (Zhao L. et al., 2024). Currently, SOX9-targeting CRISPR systems are widely utilized in scientific research, though clinical trials have not yet been initiated.

### 5.2 Nanoparticle delivery SOX9 system construction

A nanoparticle refers to an intentionally engineered material with a size below 100 nanometers, which exhibits unique chemical and physical properties not observed in larger materials. These distinctive characteristics enable diverse applications in medicine, engineering, and environmental remediation. Jae-Hwan Kim et al. designed poly (lactic-co-glycolic acid) (PLGA) nanoparticles to specifically enhance SOX9 gene expression in glial fibrillary acidic protein (GFAP)-immunoreactive astrocytes. Their observations demonstrated that PLGA nanoparticles encapsulating GFAP: SOX9: tdTOM reduce ischemia-induced neurological deficits and infarct volume via the prostaglandin D2 pathway (Kim et al., 2011). Additionally, lipid nanoparticles (LNPs) can deliver SOX9 siRNA to silence the SOX9 gene in cancer cells, thereby suppressing the anti-tumor immune response—particularly by impairing CD8^+^ T cell function—and ultimately inhibiting tumor growth (Zhang et al., 2025).

Nanoparticles themselves can interact with the immune system, sometimes eliciting beneficial responses such as enhanced vaccine efficacy or improved cancer immunotherapy. However, they may also cause adverse effects like harmful immunostimulation or immunosuppression, which could contribute to inflammation, autoimmune disorders, or increased susceptibility to infections. Key factors influencing these interactions include nanoparticle size, surface chemistry, and coating, with both innate and adaptive immune systems playing important roles (Sun et al., 2024).

### 5.3 Extracellular vesicle delivery SOX9 system construction

An extracellular vesicle (EV) delivery system employs natural, cell-derived nanocarriers to transport therapeutic agents—such as drugs, proteins, and genetic material—to specific cells and tissues. For instance, IL-10-positive EVs and SOX9-positive EVs have been used to promote M2 macrophage polarization and enhance cartilage matrix synthesis, respectively. Following the subsidence of inflammation, the expression of aggrecan and SOX9 in cartilage tissue was significantly elevated, suggesting potential strategies based on logic gates for the treatment of osteoarthritis (OA) (Li S. et al., 2024).

EVs offer several advantages, including high biocompatibility, low immunogenicity, and innate targeting capabilities. They can encapsulate a variety of biopharmaceuticals—such as synthetic RNA, enzymes, and monoclonal antibodies—protecting them from degradation and enabling efficient delivery to hard-to-reach tissues, including across the blood-brain barrier. However, EV-based therapies also face several challenges: production is difficult to standardize and scale, off-target delivery remains a concern, and therapeutic efficacy can be inconsistent. Maintaining EV stability during storage and transport is another obstacle, as conventional freezing methods are costly and may compromise their physical and biological integrity. Additionally, tumor-derived EVs carry potential risks such as tumorigenesis, metastasis, and promotion of angiogenesis (Mohak and Fabian, 2025).

### 5.4 As a peptide vaccine based on SOX9

Vaccination in the early stages of cancer is expected to effectively control the occurrence and development of tumors (Sellars et al., 2022). SOX9 has recently been considered a key regulator of triple-negative breast cancer (TNBC) metastasis (Ma Y. et al., 2020). HemaNandini et al. developed a new TNBC vaccine based on multi-epitope peptides based on SOX9, calculated and evaluated the immunodominant region of SOX9 protein, and used the antigenic epitopes obtained by fusion with suitable linkers (EAAAK, KK, AAY and GPGPG) and adjuvants (50S ribosomal protein L7/L12) to enhance the immunogenicity of the vaccine. The physicochemical properties and population coverage of the constructed vaccine were also predicted. At the same time, molecular docking and kinetic simulations were performed to study the interaction between the vaccine and Toll-like receptor 4 (TLR-4) to gain a deeper understanding of its stability. Finally, the designed vaccine was cloned into the pET28(+) vector for immunological simulation studies. Three CTL epitopes (GQVTYTGSY, NLPHYSPSY and AAGQGTGLY) and three HTL epitopes (NIETFDVNEFDQYLP, GLYSTFTYMNPAQRP and GISSTAATPASAGHV) were predicted and screened (Rajendran Krishnamoorthy and Karuppasamy, 2023). These results show that the vaccine designed based on SOX9 has the efficacy of triggering humoral and cellular immune responses.

### 5.5 Targeted SOX9 protein degradation

Currently, there is no mention of targeted protein degradation strategies for SOX9, but there have been related studies on SOX2, such as PS-NPs activated autophagy and promoted the autophagy degradation of SOX2 or ubiquitination and degradation via ChlA-F (Wan et al., 2024; Hua et al., 2020), Therefore, protein degradation targeting SOX9 is just around the corner.

## 6 Conclusion

In this article we focused on the central role of SOX9 in the immune microenvironment, systematically elaborating its regulatory mechanisms and functions in tumor immune escape, inflammatory diseases and tissue damage repair. Elevated SOX9 expression in tumor tissue promotes the transition of macrophages from the M1 to the M2 phenotype. This suppresses immune cells such as T and NK cells, facilitates tumor immune escape, and ultimately increases tumor malignancy. In contrast, in inflammatory diseases—including bone and joint disorders, hepatitis, and pulmonary fibrosis—SOX9 activation exerts beneficial effects by enhancing macrophage activity, inhibiting it pyroptosis, and promoting tissue repair, making it a promising therapeutic target. Consequently, both inhibiting SOX9 in tumors and increasing its expression in inflammatory diseases hold significant clinical potential.

### 6.1 SOX9 inhibits immune cell activity and facility tumor immune escape

Within tumor cells, SOX9 suppresses the activity, proliferation, and killing function of T cells and NK cells directly or indirectly through various mechanisms. SOX9 directly impairs T cell function by suppressing genes critical for their development and differentiation (e.g., LCK, RORC), promoting exhaustion markers (e.g., LAG3), and recruiting PMN-MDSCs via the SOX9–CXCL5 axis to inhibit lymphocyte activity. Similarly, it enables tumors to evade NK cell surveillance by modulating the expression of activating and inhibitory ligands, thereby reducing NK cell recognition and cytotoxicity. Furthermore, SOX9 influences macrophage polarization towards a pro-tumor M2 phenotype, fostering an immunosuppressive microenvironment. SOX9 strong positive correlation with key inhibitory molecules, including PD-1, PD-L1, CTLA-4, and B7-H4, highlights its role in establishing an immune-resistant niche. By directly upregulating B7-H4 via STAT3 signaling, SOX9 reduces CD8^+^ T cell infiltration while increasing Tregs, effectively shutting down anti-tumor immunity. Finally, SOX9-mediated dysregulation of chemokine signaling (e.g., reducing CXCL9/10 and CXCL16, while upregulating CXCL5) further reshapes the immune landscape by hindering the recruitment of dendritic cells and effector lymphocytes while promoting the influx of immunosuppressive MDSCs.

In conclusion, SOX9 operates as a central node in a network of immune resistance pathways. Its ability to simultaneously suppress effector immune responses and bolster immunosuppressive mechanisms makes it a highly promising therapeutic target. Future research should focus on elucidating its effects on less-studied immune cells like B cells and mast cells, and exploring the translational potential of targeting SOX9 or its downstream effectors (e.g., the SOX9–B7-H4 or SOX9–CXCL5 axes) to overcome resistance to current immunotherapies.

### 6.2 SOX9 supports macrophage function and promotes the repair of damaged tissues.

The transcription factor SOX9 emerges as a critical player across multiple disease states, with its clinical relevance particularly evident in osteoarthritis (OA), tissue repair, and chronic inflammatory conditions. The inflammatory features of OA are reflected in increased synovial levels of IL-1β, IL-6 and VEGF, Chondrocytes in OA undergo hypertrophic and senescent transition; in these states, the expression of SOX9, Acan and Col2a1 is suppressed (Horvath et al., 2023; Yousif et al., 2021). SOX9 is fundamental to cartilage integrity by stimulating the production of key extracellular matrix components like type II collagen and aggrecan, while inhibiting cartilage-degrading enzymes. During tissue repair, SOX9 acts as a regenerative mediator. Following kidney injury, SOX9 enables renal epithelial cells to produce reparative factors that influence macrophage behavior and facilitate recovery. In lung injury models, IL-4-induced SOX9 activation promotes alveolar regeneration, suggesting that augmenting SOX9 function could improve recovery in acute organ injury. Therapeutics designed to modulate SOX9—especially in coordination with immune signals—may enhance repair processes in damaged tissues. SOX9 is also implicated in chronic inflammatory and autoimmune disorders such as rheumatoid arthritis (RA), inflammatory bowel disease (IBD), and Sjögren’s syndrome.

Notably, SOX9’s role in inflammatory and degenerative diseases contrasts with its pro-tumor function in cancers. In non-malignant contexts, it often promotes tissue maintenance and repair, highlighting its therapeutic potential. Future efforts might include small molecule agents, monoclonal antibodies, or gene-based strategies to either activate or inhibit SOX9, depending on the clinical context. In summary, SOX9 represents a multifunctional regulator with significant clinical implications. Targeting its activity offers a promising approach for treating OA, enhancing tissue regeneration, and modulating immune-related diseases, paving the way for novel therapeutic strategies in precision medicine.

### 6.3 The therapeutic potential of SOX9 in immune diseases

SOX9 is a central transcriptional regulator with dualistic roles in disease, functioning as an oncogene in numerous cancers and a pro-regenerative factor in inflammatory and degenerative conditions, making it a compelling yet complex therapeutic target. In oncology, SOX9 promotes tumor persistence, drug resistance, and immune evasion through key pathways such as PI3K/AKT, Wnt/β-catenin, and TGFβ/Smad, particularly in gastric, liver, and breast cancers, where it supports cell dormancy and helps evade NK cell surveillance. It also indirectly modulates immune checkpoint molecules like PD-L1, influencing responses to immunotherapy. Conversely, in conditions like osteoarthritis and acute kidney injury, SOX9 enhances cartilage matrix synthesis, mitigates inflammation, and supports tissue repair, often by modulating macrophage activity toward an M2-polarized state. To leverage these diverse functions, multiple targeting strategies have been developed. CRISPR/Cas9 systems enable precise genetic manipulation. One strategy is to knock out SOX9, which disrupts tumor immunity and dormancy. Alternatively, SOX9 can be activated using dCas9-based systems to promote chondrogenesis and cartilage repair. These applications have been demonstrated in preclinical osteoarthritis models. Nanoparticle platforms offer versatile delivery options: PLGA nanoparticles encapsulating SOX9 plasmids enhance astrocytic SOX9 expression and reduce ischemic brain damage via the prostaglandin D2 pathway, while lipid nanoparticles (LNPs) delivering SOX9 siRNA silence its expression in tumors, countering immune evasion and inhibiting growth, though nanoparticles themselves may elicit unpredictable immune effects. While EVs face challenges in production scalability and consistency, they offer a biocompatible delivery platform for SOX9 or biologics such as IL-10. Their inherent targeting capabilities enable them to promote cartilage regeneration and macrophage polarization. For triple-negative breast cancer, multi-epitope vaccines targeting SOX9 have been designed to induce potent cellular and humoral immunity. Computationally validated to have strong TLR4 interactions, these vaccines show promise for early cancer vaccination. While targeted protein degradation of SOX9 remains unexplored, lessons from SOX2 degradation via autophagy or ubiquitination suggest a promising future for chemical degraders. Therapeutic strategies for SOX9 are entirely context-dependent. In malignancies, the goal is inhibition, whereas regeneration requires its activation. Both approaches depend on integration with advanced delivery technologies. Future efforts must prioritize cell-specific targeting, combination approaches, and translational validation to fully exploit SOX9’s clinical potential.

### 6.4 Research deficiencies and prospects

Unfortunately, there is still limited research on SOX9 in the field of immunology, and many detailed mechanisms of immune regulation remain unclear. Our understanding of the SOX9 regulatory network is deepening, and drug development technologies are advancing. On one hand, these enable therapeutic strategies that target SOX9. These include inhibitors for cancer immunotherapy and agonists for regenerative medicine, both demonstrating broad clinical prospects. On the other hand, their ultimate success depends on overcoming key challenges. Specifically, we must resolve issues of tissue-specific delivery and ensure treatment safety.

## Glossary

SOX9: Sex-determining region Y-related high-mobility group box 9
TFs: Transcription factors
ACT: Adoptive cell transfer
CAR-T: Chimeric antigen receptor T-cell
CTLA-4: Cytotoxic T-lymphocyte-associated protein 4
PD-1: Programmed cell death protein 1
HMG: High Mobility Group
TAC: C-terminal transcriptional activation domain
DIM: Dimerization domain
TAM: Transcriptional activation domain
DCs: Dendritic cells
DTCs: Disseminated tumor cells
LCC: latency-competent cancer
MDSCs: Myeloid-derived suppressor cell
CRC: Colorectal cancer
HCC: hepatocellular carcinoma
GAC: Gastric adenocarcinoma
PMN-MDSCs: Polymorphonuclear myeloid-derived suppressor cells
PBMCs: Peripheral blood mononuclear cells
NK: Natural killer
RA: Retinoic acid
LIF: Leukaemia inhibitory factor
PLGA: Poly lactic-co-glycolic acid
GFAP: Glial fibrillary acidic protein
sgRNA: Single-guide RNA
ATIIs: Alveolar epithelial cells
ZFP24: zinc finger protein 24
NR4A1: Nuclear receptor subfamily 4 group A
OA: Osteoarthritis
ECM: Extracellular matrix
AKI: Acute kidney injury
IRI: Ischemia-reperfusion injury
TNF-α: Tumor necrosis factor α
SLE: Systemic lupus erythematosus
IBD: Inflammatory bowel disease
TNBC: Triple-negative breast cancer
TLR-4: Toll-like receptor 4
ULBPs: UL16-binding proteins
LNPs: Lipid nanoparticles
EVs: Extracellular vesicles.

## References

[B1] Adusumilli P. S. Zauderer M. G. Riviere I. Solomon S. B. Rusch V. W. O'Cearbhaill R. E. (2021). A phase I trial of regional mesothelin-targeted CAR T-cell therapy in patients with malignant pleural disease, in combination with the Anti-PD-1 agent pembrolizumab. Cancer Discov. 11, 2748–2763. 10.1158/2159-8290.CD-21-0407 34266984 PMC8563385

[B2] Aggarwal S. Wang Z. Rincon Fernandez Pacheco D. Rinaldi A. Rajewski A. Callemeyn J. (2024). SOX9 switch links regeneration to fibrosis at the single-cell level in mammalian kidneys. Science 383, eadd6371. 10.1126/science.add6371 38386758 PMC11345873

[B3] Akiyama H. Lyons J. P. Mori-Akiyama Y. Yang X. Zhang R. Zhang Z. (2004). Interactions between Sox9 and beta-catenin control chondrocyte differentiation. Genes Dev. 18, 1072–1087. 10.1101/gad.1171104 15132997 PMC406296

[B4] Argentaro A. Sim H. Kelly S. Preiss S. Clayton A. Jans D. A. (2003). A SOX9 defect of calmodulin-dependent nuclear import in campomelic dysplasia/autosomal sex reversal. J. Biol. Chem. 278, 33839–33847. 10.1074/jbc.M302078200 12810722

[B5] Ashrafizadeh M. Zarrabi A. Orouei S. Zabolian A. Saleki H. Azami N. (2021). Interplay between SOX9 transcription factor and microRNAs in cancer. Int. J. Biol. Macromol. 183, 681–694. 10.1016/j.ijbiomac.2021.04.185 33957202

[B6] Bell D. M. Leung K. K. Wheatley S. C. Ng L. J. Zhou S. Ling K. W. (1997). SOX9 directly regulates the type-II collagen gene. Nat. Genet. 16, 174–178. 10.1038/ng0697-174 9171829

[B7] Bian X. Wang W. Abudurexiti M. Zhang X. Ma W. Shi G. (2024). Integration analysis of single-cell multi-omics reveals prostate cancer heterogeneity. Adv. Sci. (Weinh) 11, e2305724. 10.1002/advs.202305724 38483933 PMC11095148

[B8] Borgenvik A. Holmberg K. O. Bolin S. Zhao M. Savov V. Rosen G. (2022). Dormant SOX9-Positive cells facilitate MYC-Driven recurrence of medulloblastoma. Cancer Res. 82, 4586–4603. 10.1158/0008-5472.CAN-22-2108 36219398 PMC9755969

[B9] Cai X. T. Jia M. Heigl T. Shamir E. R. Wong A. K. Hall B. M. (2024). IL-4-induced SOX9 confers lineage plasticity to aged adult lung stem cells. Cell Rep. 43, 114569. 10.1016/j.celrep.2024.114569 39088319

[B10] Chen Y. Kim J. Yang S. Wang H. Wu C. J. Sugimoto H. (2021). Type I collagen deletion in alphaSMA(+) myofibroblasts augments immune suppression and accelerates progression of pancreatic cancer. Cancer Cell 39, 548–565 e546. 10.1016/j.ccell.2021.02.007 33667385 PMC8423173

[B11] Chen J. W. Huang M. J. Chen X. N. Wu L. L. Li Q. G. Hong Q. (2022). Transient upregulation of EGR1 signaling enhances kidney repair by activating SOX9(+) renal tubular cells. Theranostics 12, 5434–5450. 10.7150/thno.73426 35910788 PMC9330523

[B12] Chen Q. Weng K. Lin M. Jiang M. Fang Y. Chung S. S. W. (2023). SOX9 modulates the transformation of gastric stem cells through biased symmetric cell division. Gastroenterology 164, 1119–1136.e12. 10.1053/j.gastro.2023.01.037 36740200 PMC10200757

[B13] Christin J. R. Wang C. Chung C. Y. Liu Y. Dravis C. Tang W. (2020). Stem cell determinant SOX9 promotes lineage plasticity and progression in basal-like breast cancer. Cell Rep. 31, 107742. 10.1016/j.celrep.2020.107742 32521267 PMC7658810

[B14] Demaria O. Cornen S. Daeron M. Morel Y. Medzhitov R. Vivier E. (2019). Harnessing innate immunity in cancer therapy. Nature 574, 45–56. 10.1038/s41586-019-1593-5 31578484

[B15] Du T. Y. Gao Y. X. Zheng Y. S. (2023). Identification of key genes related to immune infiltration in cirrhosis *via* bioinformatics analysis. Sci. Rep. 13, 1876. 10.1038/s41598-022-26794-8 36725885 PMC9892033

[B16] Faleeva M. Ahmad S. Theofilatos K. Lynham S. Watson G. Whitehead M. (2024). Sox9 accelerates vascular aging by regulating extracellular matrix composition and stiffness. Circ. Res. 134, 307–324. 10.1161/CIRCRESAHA.123.323365 38179698 PMC10826924

[B17] Fan Y. Li Y. Yao X. Jin J. Scott A. Liu B. (2023). Epithelial SOX9 drives progression and metastases of gastric adenocarcinoma by promoting immunosuppressive tumour microenvironment. Gut 72, 624–637. 10.1136/gutjnl-2021-326581 36002248

[B18] Fang C. Zhong R. Lu S. Yu G. Liu Z. Yan C. (2024). TREM2 promotes macrophage polarization from M1 to M2 and suppresses osteoarthritis through the NF-κB/CXCL3 axis. Int. J. Biol. Sci. 20, 1992–2007. 10.7150/ijbs.91519 38617547 PMC11008261

[B19] Feng D. Xiang X. Guan Y. Guillot A. Lu H. Chang C. (2023). Monocyte-derived macrophages orchestrate multiple cell-type interactions to repair necrotic liver lesions in disease models. J. Clin. Invest 133, e166954. 10.1172/JCI166954 37338984 PMC10378165

[B20] Feng Q. Cui N. Li S. Cao J. Chen Q. Wang H. (2023). Upregulation of SOX9 promotes the self-renewal and tumorigenicity of cervical cancer through activating the Wnt/β-catenin signaling pathway. FASEB J. 37, e23174. 10.1096/fj.202201596RRR 37668416

[B21] Galli E. Bellesi S. Pansini I. Di Cesare G. Iacovelli C. Malafronte R. (2023). The CD4/CD8 ratio of infused CD19-CAR-T is a prognostic factor for efficacy and toxicity. Br. J. Haematol. 203, 564–570. 10.1111/bjh.19117 37789569

[B22] Geraldo M. T. Valente G. T. Nakajima R. T. Martins C. (2016). Dimerization and transactivation domains as candidates for functional modulation and diversity of Sox9. PLoS One 11, e0156199. 10.1371/journal.pone.0156199 27196604 PMC4873142

[B23] Gonzalez-Rodriguez S. Lorenzo-Herrero S. Sordo-Bahamonde C. Hidalgo A. Gonzalez S. Menendez L. (2022). Involvement of CD4(+) and CD8(+) T-lymphocytes in the modulation of nociceptive processing evoked by CCL4 in mice. Life Sci. 291, 120302. 10.1016/j.lfs.2022.120302 34999112

[B24] Grimm D. Bauer J. Wise P. Kruger M. Simonsen U. Wehland M. (2020). The role of SOX family members in solid tumours and metastasis. Semin. Cancer Biol. 67, 122–153. 10.1016/j.semcancer.2019.03.004 30914279

[B25] Guilliams M. Scott C. L. (2022). Liver macrophages in health and disease. Immunity 55, 1515–1529. 10.1016/j.immuni.2022.08.002 36103850

[B26] Guo Y. Cen K. Hong K. Mai Y. Jiang M. (2023). Construction of a neural network diagnostic model for renal fibrosis and investigation of immune infiltration characteristics. Front. Immunol. 14, 1183088. 10.3389/fimmu.2023.1183088 37359552 PMC10288286

[B27] Haseeb A. Lefebvre V. (2019). The SOXE transcription factors-SOX8, SOX9 and SOX10-share a bi-partite transactivation mechanism. Nucleic Acids Res. 47, 6917–6931. 10.1093/nar/gkz523 31194875 PMC6649842

[B28] He S. Feng Y. Zou W. Wang J. Li G. Xiong W. (2021). The role of the SOX9/lncRNA ANXA2P2/miR-361-3p/SOX9 regulatory loop in cervical cancer cell growth and resistance to cisplatin. Front. Oncol. 11, 784525. 10.3389/fonc.2021.784525 35083143 PMC8784813

[B29] Horvath E. Solyom A. Szekely J. Nagy E. E. Popoviciu H. (2023). Inflammatory and metabolic signaling interfaces of the hypertrophic and senescent chondrocyte phenotypes associated with osteoarthritis. Int. J. Mol. Sci. 24, 16468. 10.3390/ijms242216468 38003658 PMC10671750

[B30] Hua X. Huang M. Deng X. Xu J. Luo Y. Xie Q. (2020). The inhibitory effect of compound ChlA-F on human bladder cancer cell invasion can be attributed to its blockage of SOX2 protein. Cell Death Differ. 27, 632–645. 10.1038/s41418-019-0377-7 31243344 PMC7205984

[B31] Huang W. Lu N. Eberspaecher H. De Crombrugghe B. (2002). A new long form of c-Maf cooperates with Sox9 to activate the type II collagen gene. J. Biol. Chem. 277, 50668–50675. 10.1074/jbc.M206544200 12381733

[B32] Imagawa K. de Andres M. C. Hashimoto K. Itoi E. Otero M. Roach H. I. (2014). Association of reduced type IX collagen gene expression in human osteoarthritic chondrocytes with epigenetic silencing by DNA hypermethylation. Arthritis Rheumatol. 66, 3040–3051. 10.1002/art.38774 25048791 PMC4211984

[B33] Jana S. Madhu Krishna B. Singhal J. Horne D. Awasthi S. Salgia R. (2020). SOX9: the master regulator of cell fate in breast cancer. Biochem. Pharmacol. 174, 113789. 10.1016/j.bcp.2019.113789 31911091 PMC9048250

[B34] Jeong S. Y. Kang M. L. Park J. W. Im G. I. (2020). Dual functional nanoparticles containing SOX duo and ANGPT4 shRNA for osteoarthritis treatment. J. Biomed. Mater Res. B Appl. Biomater. 108, 234–242. 10.1002/jbm.b.34383 30957437

[B35] Jiang B. C. Liu T. Gao Y. J. (2020). Chemokines in chronic pain: cellular and molecular mechanisms and therapeutic potential. Pharmacol. Ther. 212, 107581. 10.1016/j.pharmthera.2020.107581 32450191

[B36] Kawaguchi Y. (2013). Sox9 and programming of liver and pancreatic progenitors. J. Clin. Invest 123, 1881–1886. 10.1172/JCI66022 23635786 PMC3635727

[B37] Kim J. H. Park J. S. Yang H. N. Woo D. G. Jeon S. Y. Do H. J. (2011). The use of biodegradable PLGA nanoparticles to mediate SOX9 gene delivery in human mesenchymal stem cells (hMSCs) and induce chondrogenesis. Biomaterials 32, 268–278. 10.1016/j.biomaterials.2010.08.086 20875683

[B38] Kim J. Y. Silvaroli J. A. Martinez G. V. Bisunke B. Luna Ramirez A. V. Jayne L. A. (2023). Zinc finger protein 24-dependent transcription factor SOX9 up-regulation protects tubular epithelial cells during acute kidney injury. Kidney Int. 103, 1093–1104. 10.1016/j.kint.2023.02.026 36921719 PMC10200760

[B39] Kobayashi M. Usui T. Elbadawy M. Kigata T. Kaneda M. Murakami T. (2024). The increase in the frequency and amplitude of the beating of isolated mouse tracheal cilia reactivated by ATP and cAMP with elevation in pH. Int. J. Mol. Sci. 25, 8138. 10.3390/ijms25158138 39125708 PMC11312401

[B40] Kumar S. (2018). Cellular and molecular pathways of renal repair after acute kidney injury. Kidney Int. 93, 27–40. 10.1016/j.kint.2017.07.030 29291820

[B41] Lefebvre V. Angelozzi M. Haseeb A. (2019). SOX9 in cartilage development and disease. Curr. Opin. Cell Biol. 61, 39–47. 10.1016/j.ceb.2019.07.008 31382142 PMC6956855

[B42] Li J. Lee Y. Li Y. Jiang Y. Lu H. Zang W. (2018). Co-inhibitory molecule B7 superfamily member 1 expressed by tumor-infiltrating myeloid cells induces dysfunction of anti-tumor CD8(+) T cells. Immunity 48, 773–786.e5. 10.1016/j.immuni.2018.03.018 29625896

[B43] Li G. Fang Y. Xu N. Ding Y. Liu D. (2024). Fibroblast-like synoviocytes-derived exosomal circFTO deteriorates rheumatoid arthritis by enhancing N6-methyladenosine modification of SOX9 in chondrocytes. Arthritis Res. Ther. 26, 56. 10.1186/s13075-024-03290-0 38388473 PMC10882813

[B44] Li S. Zheng W. Deng W. Li Z. Yang J. Zhang H. (2024). Logic-based strategy for spatiotemporal release of dual extracellular vesicles in osteoarthritis treatment. Adv. Sci. (Weinh) 11, e2403227. 10.1002/advs.202403227 38704731 PMC11234466

[B45] Liu S. Galat V. Galat Y. Lee Y. K. A. Wainwright D. Wu J. (2021). NK cell-based cancer immunotherapy: from basic biology to clinical development. J. Hematol. Oncol. 14, 7. 10.1186/s13045-020-01014-w 33407739 PMC7788999

[B46] Liu Y. Zhuo S. Zhou Y. Ma L. Sun Z. Wu X. (2022). Yap-Sox9 signaling determines hepatocyte plasticity and lineage-specific hepatocarcinogenesis. J. Hepatol. 76, 652–664. 10.1016/j.jhep.2021.11.010 34793870 PMC8858854

[B47] Liu Z. Liu J. Chen T. Wang Y. Shi A. Li K. (2022). Wnt-TCF7-SOX9 axis promotes cholangiocarcinoma proliferation and pemigatinib resistance in a FGF7-FGFR2 autocrine pathway. Oncogene 41, 2885–2896. 10.1038/s41388-022-02313-x 35428876

[B48] Liu Y. John P. Nishitani K. Cui J. Nishimura C. D. Christin J. R. (2023). A SOX9-B7x axis safeguards dedifferentiated tumor cells from immune surveillance to drive breast cancer progression. Dev. Cell 58, 2700–2717.e12. 10.1016/j.devcel.2023.10.010 37963469 PMC10842074

[B49] Liu S. Yang L. Fu J. Li T. Zhou B. Wang K. (2023). Comprehensive analysis, immune, and cordycepin regulation for SOX9 expression in pan-cancers and the matched healthy tissues. Front. Immunol. 14, 1149986. 10.3389/fimmu.2023.1149986 37020558 PMC10067558

[B50] Liu J. A. Tam K. W. Chen Y. L. Feng X. Chan C. W. L. Lo A. L. H. (2023). Transplanting human neural stem cells with ≈50% reduction of SOX9 gene dosage promotes tissue repair and functional recovery from severe spinal cord injury. Adv. Sci. (Weinh) 10, e2205804. 10.1002/advs.202205804 37296073 PMC10369238

[B51] Luo H. Wang C. Liu M. Yin B. A P. Huang D. (2018). Inhibition of SOX9 promotes inflammatory and immune responses of dental pulp. J. Endod. 44, 792–799. 10.1016/j.joen.2018.02.004 29571909

[B52] Luo X. Ji X. Xie M. Zhang T. Wang Y. Sun M. (2022). Advance of SOX transcription factors in hepatocellular carcinoma: from role, tumor immune relevance to targeted therapy. Cancers (Basel) 14, 1165. 10.3390/cancers14051165 35267473 PMC8909699

[B53] Ma X. L. Hu B. Tang W. G. Xie S. H. Ren N. Guo L. (2020). CD73 sustained cancer-stem-cell traits by promoting SOX9 expression and stability in hepatocellular carcinoma. J. Hematol. Oncol. 13, 11. 10.1186/s13045-020-0845-z 32024555 PMC7003355

[B54] Ma Y. Shepherd J. Zhao D. Bollu L. R. Tahaney W. M. Hill J. (2020). SOX9 is essential for triple-negative breast cancer cell survival and metastasis. Mol. Cancer Res. 18, 1825–1838. 10.1158/1541-7786.MCR-19-0311 32661114 PMC7718423

[B55] Ma H. Siu W. S. Koon C. M. Wu X. X. Li X. Cheng W. (2023). The application of adipose tissue-derived mesenchymal stem cells (ADMSCs) and a twin-herb formula to the rodent wound healing model: use alone or together? Int. J. Mol. Sci. 24, 1372. 10.3390/ijms24021372 36674885 PMC9867064

[B56] Malladi S. Macalinao D. G. Jin X. He L. Basnet H. Zou Y. (2016). Metastatic latency and immune evasion through autocrine inhibition of WNT. Cell 165, 45–60. 10.1016/j.cell.2016.02.025 27015306 PMC4808520

[B57] Mantovani A. Garlanda C. (2023). Humoral innate immunity and acute-phase proteins. N. Engl. J. Med. 388, 439–452. 10.1056/NEJMra2206346 36724330 PMC9912245

[B58] Martel-Pelletier J. Barr A. J. Cicuttini F. M. Conaghan P. G. Cooper C. Goldring M. B. (2016). Osteoarthritis. Nat. Rev. Dis. Prim. 2, 16072. 10.1038/nrdp.2016.72 27734845

[B59] McKillop W. M. Dragan M. Schedl A. Brown A. (2013). Conditional Sox9 ablation reduces chondroitin sulfate proteoglycan levels and improves motor function following spinal cord injury. Glia 61, 164–177. 10.1002/glia.22424 23027386 PMC4853194

[B60] Mei Z. Yilamu K. Ni W. Shen P. Pan N. Chen H. (2025). Chondrocyte fatty acid oxidation drives osteoarthritis *via* SOX9 degradation and epigenetic regulation. Nat. Commun. 16, 4892. 10.1038/s41467-025-60037-4 40425566 PMC12117060

[B61] Meng X. Zhu H. (2023). SOX9 inhibits the progression of osteonecrosis of the femoral head *via* the activation of the Wnt/beta-catenin pathway. J. Invest Surg. 36, 2197054. 10.1080/08941939.2023.2197054 37076124

[B62] Met O. Jensen K. M. Chamberlain C. A. Donia M. Svane I. M. (2019). Principles of adoptive T cell therapy in cancer. Semin. Immunopathol. 41, 49–58. 10.1007/s00281-018-0703-z 30187086

[B63] Michelatti D. Beyes S. Bernardis C. Negri M. L. Morelli L. Bediaga N. G. (2024). Oncogenic enhancers prime quiescent metastatic cells to escape NK immune surveillance by eliciting transcriptional memory. Nat. Commun. 15, 2198. 10.1038/s41467-024-46524-0 38503727 PMC10951355

[B64] Mochizuki Y. Chiba T. Kataoka K. Yamashita S. Sato T. Kato T. (2018). Combinatorial CRISPR/Cas9 approach to elucidate a far-upstream enhancer complex for tissue-specific Sox9 expression. Dev. Cell 46, 794–806.e6. 10.1016/j.devcel.2018.07.024 30146478 PMC6324936

[B65] Mohak S. Fabian Z. (2025). Extracellular vesicles as precision delivery systems for biopharmaceuticals: innovations, challenges, and therapeutic potential. Pharmaceutics 17, 641. 10.3390/pharmaceutics17050641 40430932 PMC12115175

[B66] Mughees M. Kaushal J. B. Sharma G. Wajid S. Batra S. K. Siddiqui J. A. (2022). Chemokines and cytokines: axis and allies in prostate cancer pathogenesis. Semin. Cancer Biol. 86, 497–512. 10.1016/j.semcancer.2022.02.017 35181473 PMC9793433

[B67] Natarajan P. Koupourtidou C. de Resseguier T. Thorwirth M. Bocchi R. Fischer-Sternjak J. (2025). Single cell deletion of the transcription factors Trps1 and Sox9 in astrocytes reveals novel functions in the adult cerebral cortex. Glia 73, 737–758. 10.1002/glia.24645 39610085 PMC11845849

[B68] Neo S. Y. Jing X. Tong L. Tong D. Gao J. Chen Z. (2022). Tumor MHC class I expression alters cancer-associated myelopoiesis driven by host NK cells. J. Immunother. Cancer 10, e005308. 10.1136/jitc-2022-005308 36283735 PMC9608525

[B69] Nie H. Zhao Z. Zhou D. Li D. Wang Y. Ma Y. (2023). Activated SOX9+ renal epithelial cells promote kidney repair through secreting factors. Cell Prolif. 56, e13394. 10.1111/cpr.13394 36601693 PMC10068929

[B70] Olsen R. R. Ireland A. S. Kastner D. W. Groves S. M. Spainhower K. B. Pozo K. (2021). ASCL1 represses a SOX9(+) neural crest stem-like state in small cell lung cancer. Genes Dev. 35, 847–869. 10.1101/gad.348295.121 34016693 PMC8168563

[B71] Panda M. Tripathi S. K. Biswal B. K. (2021). SOX9: an emerging driving factor from cancer progression to drug resistance. Biochim. Biophys. Acta Rev. Cancer 1875, 188517. 10.1016/j.bbcan.2021.188517 33524528

[B72] Park Y. Hu S. Kim M. Oertel M. Singhi A. Monga S. P. (2024). Context-dependent distinct roles of SOX9 in combined hepatocellular carcinoma-cholangiocarcinoma. Cells 13 (17), 1451. 10.3390/cells13171451 39273023 PMC11394107

[B73] Parker M. E. Ciofani M. (2020). Regulation of γδ T cell effector diversification in the thymus. Front. Immunol. 11, 42. 10.3389/fimmu.2020.00042 32038664 PMC6992645

[B74] Parkin J. Cohen B. (2001). An overview of the immune system. Lancet 357, 1777–1789. 10.1016/s0140-6736(00)04904-7 11403834

[B75] Postow M. A. Sidlow R. Hellmann M. D. (2018). Immune-related adverse events associated with immune checkpoint blockade. N. Engl. J. Med. 378, 158–168. 10.1056/NEJMra1703481 29320654

[B76] Pulanco M. C. Madsen A. T. Tanwar A. Corrigan D. T. Zang X. (2023). Recent advancements in the B7/CD28 immune checkpoint families: new biology and clinical therapeutic strategies. Cell Mol. Immunol. 20, 694–713. 10.1038/s41423-023-01019-8 37069229 PMC10310771

[B77] Qi G. Li L. (2020). Long non-coding RNA PVT1 contributes to cell growth and metastasis in non-small-cell lung cancer by regulating miR-361-3p/SOX9 axis and activating Wnt/β-catenin signaling pathway. Biomed. Pharmacother. 126, 110100. 10.1016/j.biopha.2020.110100 32197208

[B78] Qian H. Ding C. H. Liu F. Chen S. J. Huang C. K. Xiao M. C. (2024). SRY-box transcription factor 9 triggers YAP nuclear entry *via* direct interaction in tumors. Signal Transduct. Target Ther. 9, 96. 10.1038/s41392-024-01805-4 38653754 PMC11039692

[B79] Rajendran Krishnamoorthy H. Karuppasamy R. (2023). Designing a novel SOX9 based multi-epitope vaccine to combat metastatic triple-negative breast cancer using immunoinformatics approach. Mol. Divers 27, 1829–1842. 10.1007/s11030-022-10539-w 36214961 PMC9549049

[B80] Ramakrishnan A. B. Burby P. E. Adiga K. Cadigan K. M. (2023). SOX9 and TCF transcription factors associate to mediate Wnt/β-catenin target gene activation in colorectal cancer. J. Biol. Chem. 299, 102735. 10.1016/j.jbc.2022.102735 36423688 PMC9771724

[B81] Ren Z. Chen Y. Shi L. Shao F. Sun Y. Ge J. (2022). Sox9/CXCL5 axis facilitates tumour cell growth and invasion in hepatocellular carcinoma. FEBS J. 289, 3535–3549. 10.1111/febs.16357 35038357

[B82] Ribas A. Wolchok J. D. (2018). Cancer immunotherapy using checkpoint blockade. Science 359, 1350–1355. 10.1126/science.aar4060 29567705 PMC7391259

[B83] Rowshanravan B. Halliday N. Sansom D. M. (2018). CTLA-4: a moving target in immunotherapy. Blood 131, 58–67. 10.1182/blood-2017-06-741033 29118008 PMC6317697

[B84] Saunders P. M. Vivian J. P. O'Connor G. M. Sullivan L. C. Pymm P. Rossjohn J. (2015). A bird's eye view of NK cell receptor interactions with their MHC class I ligands. Immunol. Rev. 267, 148–166. 10.1111/imr.12319 26284476

[B85] Sellars M. C. Wu C. J. Fritsch E. F. (2022). Cancer vaccines: building a bridge over troubled waters. Cell 185, 2770–2788. 10.1016/j.cell.2022.06.035 35835100 PMC9555301

[B86] Shen Y. Zhou J. Nie K. Cheng S. Chen Z. Wang W. (2022). Oncogenic role of the SOX9-DHCR24-cholesterol biosynthesis axis in IGH-BCL2+ diffuse large B-cell lymphomas. Blood 139, 73–86. 10.1182/blood.2021012327 34624089 PMC8740888

[B87] Sheng M. Weng Y. Cao Y. Zhang C. Lin Y. Yu W. (2023). Caspase 6/NR4A1/SOX9 signaling axis regulates hepatic inflammation and pyroptosis in ischemia-stressed fatty liver. Cell Death Discov. 9, 106. 10.1038/s41420-023-01396-z 36977670 PMC10043527

[B88] Shimasaki N. Jain A. Campana D. (2020). NK cells for cancer immunotherapy. Nat. Rev. Drug Discov. 19, 200–218. 10.1038/s41573-019-0052-1 31907401

[B89] Shin H. J. Choi S. G. Qu F. Yi M. H. Lee C. H. Kim S. R. (2024). Peptide-mediated targeted delivery of SOX9 nanoparticles into astrocytes ameliorates ischemic brain injury. Nanoscale 16, 833–847. 10.1039/d3nr01318a 38093712

[B90] Sterner R. C. Sterner R. M. (2021). CAR-T cell therapy: current limitations and potential strategies. Blood Cancer J. 11, 69. 10.1038/s41408-021-00459-7 33824268 PMC8024391

[B91] Subhadarshini S. Sahoo S. Debnath S. Somarelli J. A. Jolly M. K. (2023). Dynamical modeling of proliferative-invasive plasticity and IFNgamma signaling in melanoma reveals mechanisms of PD-L1 expression heterogeneity. J. Immunother. Cancer 11 (9), e006766. 10.1136/jitc-2023-006766 37678920 PMC10496669

[B92] Sun Q. Zhuang Z. Bai R. Deng J. Xin T. Zhang Y. (2023). Lysine 68 methylation-dependent SOX9 stability control modulates chondrogenic differentiation in dental pulp stem cells. Adv. Sci. (Weinh) 10, e2206757. 10.1002/advs.202206757 37386801 PMC10460901

[B93] Sun Y. Liu Y. Li R. Zhang C. Wu M. Zhang X. (2024). Multifunctional biomimetic nanocarriers for dual-targeted immuno-gene therapy against hepatocellular carcinoma. Adv. Sci. (Weinh) 11, e2400951. 10.1002/advs.202400951 38973319 PMC11425963

[B94] Terren I. Orrantia A. Vitalle J. Zenarruzabeitia O. Borrego F. (2019). NK cell metabolism and tumor microenvironment. Front. Immunol. 10, 2278. 10.3389/fimmu.2019.02278 31616440 PMC6769035

[B95] Tian B. Zhang L. Zheng J. Kang X. (2024). The role of NF-κB-SOX9 signalling pathway in osteoarthritis. Heliyon 10, e37191. 10.1016/j.heliyon.2024.e37191 39319133 PMC11419907

[B96] Topalian S. L. Taube J. M. Anders R. A. Pardoll D. M. (2016). Mechanism-driven biomarkers to guide immune checkpoint blockade in cancer therapy. Nat. Rev. Cancer 16, 275–287. 10.1038/nrc.2016.36 27079802 PMC5381938

[B97] Tripathi S. K. Sahoo R. K. Biswal B. K. (2022). SOX9 as an emerging target for anticancer drugs and a prognostic biomarker for cancer drug resistance. Drug Discov. Today 27, 2541–2550. 10.1016/j.drudis.2022.05.022 35636723

[B98] Tsuchiya M. Ogawa H. Suzuki T. Sugiyama N. Haraguchi T. Hiraoka Y. (2011). Exportin 4 interacts with Sox9 through the HMG box and inhibits the DNA binding of Sox9. PLoS One 6, e25694. 10.1371/journal.pone.0025694 21991335 PMC3185033

[B99] Underwood A. Rasicci D. T. Hinds D. Mitchell J. T. Zieba J. K. Mills J. (2023). Evolutionary landscape of SOX genes to inform genotype-to-phenotype relationships. Genes (Basel) 14, 222. 10.3390/genes14010222 36672963 PMC9859272

[B100] van Beuningen H. M. de Vries-van Melle M. L. Vitters E. L. Schreurs W. van den Berg W. B. van Osch G. J. (2014). Inhibition of TAK1 And/or JAK can rescue impaired chondrogenic differentiation of human mesenchymal stem cells in osteoarthritis-like conditions. Tissue Eng. Part A 20, 2243–2252. 10.1089/ten.TEA.2013.0553 24547725 PMC4137338

[B101] Vilgelm A. E. Richmond A. (2019). Chemokines modulate immune surveillance in tumorigenesis, metastasis, and response to immunotherapy. Front. Immunol. 10, 333. 10.3389/fimmu.2019.00333 30873179 PMC6400988

[B102] Wan S. Wang X. Chen W. Xu Z. Zhao J. Huang W. (2024). Polystyrene nanoplastics activate autophagy and suppress trophoblast cell migration/invasion and migrasome formation to induce miscarriage. ACS Nano 18, 3733–3751. 10.1021/acsnano.3c11734 38252510

[B103] Wang L. Zhang Z. Yu X. Li Q. Wang Q. Chang A. (2020). SOX9/miR-203a axis drives PI3K/AKT signaling to promote esophageal cancer progression. Cancer Lett. 468, 14–26. 10.1016/j.canlet.2019.10.004 31600529

[B104] Wang M. Wang Z. Zhi X. Ding W. Xiong J. Tao T. (2020). SOX9 enhances sorafenib resistance through upregulating ABCG2 expression in hepatocellular carcinoma. Biomed. Pharmacother. 129, 110315. 10.1016/j.biopha.2020.110315 32554246

[B105] Wang C. Xue W. Zhang H. Fu Y. (2021). Identification of candidate genes encoding tumor-specific neoantigens in early- and late-stage colon adenocarcinoma. Aging (Albany NY) 13, 4024–4044. 10.18632/aging.202370 33428592 PMC7906157

[B106] Watmough S. A. (2024). Critical loads for alkalization in terrestrial ecosystems. Sci. Total Environ. 927, 171967. 10.1016/j.scitotenv.2024.171967 38537833

[B107] Wu C. Huang Z. Chen J. Li N. Cai Y. Chen J. (2025). Efficiently directing differentiation and homing of mesenchymal stem cells to boost cartilage repair in osteoarthritis *via* a nanoparticle and peptide dual-engineering strategy. Biomaterials 312, 122720. 10.1016/j.biomaterials.2024.122720 39084098

[B108] Wynn T. A. Vannella K. M. (2016). Macrophages in tissue repair, regeneration, and fibrosis. Immunity 44, 450–462. 10.1016/j.immuni.2016.02.015 26982353 PMC4794754

[B109] Xiang N. Xu H. Zhou Z. Wang J. Cai P. Wang L. (2023). Single-cell transcriptome profiling reveals immune and stromal cell heterogeneity in primary sjögren's syndrome. iScience 26, 107943. 10.1016/j.isci.2023.107943 37810210 PMC10558796

[B110] Xie M. Wu Z. Ying S. Liu L. Zhao C. Yao C. (2021). Sublytic C5b-9 induces glomerular mesangial cell proliferation *via* ERK1/2-dependent SOX9 phosphorylation and acetylation by enhancing cyclin D1 in rat Thy-1 nephritis. Exp. Mol. Med. 53, 572–590. 10.1038/s12276-021-00589-9 33811247 PMC8102557

[B111] Xue Y. Lian W. Zhi J. Yang W. Li Q. Guo X. (2019). HDAC5-mediated deacetylation and nuclear localisation of SOX9 is critical for tamoxifen resistance in breast cancer. Br. J. Cancer 121, 1039–1049. 10.1038/s41416-019-0625-0 31690832 PMC6964674

[B112] Yan F. Teng Y. Li X. Zhong Y. Li C. Yan F. (2024). Hypoxia promotes non-small cell lung cancer cell stemness, migration, and invasion *via* promoting glycolysis by lactylation of SOX9. Cancer Biol. Ther. 25, 2304161. 10.1080/15384047.2024.2304161 38226837 PMC10793688

[B113] Yang Y. Gomez N. Infarinato N. Adam R. C. Sribour M. Baek I. (2023). The pioneer factor SOX9 competes for epigenetic factors to switch stem cell fates. Nat. Cell Biol. 25, 1185–1195. 10.1038/s41556-023-01184-y 37488435 PMC10415178

[B114] Yang Y. Chen Y. Liu Z. Chang Z. Sun Z. Zhao L. (2024). Concomitant NAFLD facilitates liver metastases and PD-1-Refractory by recruiting MDSCs *via* CXCL5/CXCR2 in colorectal cancer. Cell Mol. Gastroenterol. Hepatol. 18, 101351. 10.1016/j.jcmgh.2024.04.008 38724007 PMC11227024

[B115] Ye X. Cen Y. Li Q. Zhang Y. P. Li Q. Li J. (2024). Immunosuppressive SOX9-AS1 resists triple-negative breast cancer senescence *via* regulating wnt signalling pathway. J. Cell Mol. Med. 28, e70208. 10.1111/jcmm.70208 39550706 PMC11569622

[B116] Yi M. Zheng X. Niu M. Zhu S. Ge H. Wu K. (2022). Combination strategies with PD-1/PD-L1 blockade: current advances and future directions. Mol. Cancer 21, 28. 10.1186/s12943-021-01489-2 35062949 PMC8780712

[B117] Yousif A. S. Ronsard L. Shah P. Omatsu T. Sangesland M. Bracamonte Moreno T. (2021). The persistence of interleukin-6 is regulated by a blood buffer system derived from dendritic cells. Immunity 54, 235–246.e5. 10.1016/j.immuni.2020.12.001 33357409 PMC7836640

[B118] Yuan X. Huang L. Luo W. Zhao Y. Nashan B. Yu F. (2021). Diagnostic and prognostic significances of SOX9 in thymic epithelial tumor. Front. Oncol. 11, 708735. 10.3389/fonc.2021.708735 34778027 PMC8580949

[B119] Zhang G. Miyamoto M. M. Cohn M. J. (2006). Lamprey type II collagen and Sox9 reveal an ancient origin of the vertebrate collagenous skeleton. Proc. Natl. Acad. Sci. U. S. A. 103, 3180–3185. 10.1073/pnas.0508313103 16492784 PMC1413883

[B120] Zhang K. Chen S. Sun H. Wang L. Li H. Zhao J. (2020). *In vivo* two-photon microscopy reveals the contribution of Sox9(+) cell to kidney regeneration in a mouse model with extracellular vesicle treatment. J. Biol. Chem. 295, 12203–12213. 10.1074/jbc.ra120.012732 32641493 PMC7443503

[B121] Zhang Z. Wang H. Zhang Z. Zhang Y. Sun H. Chen X. (2025). SOX9 siRNA loaded lipid nanoparticles actively targeted: formulation, delivery, and antitumor effect on colorectal cancer *in vitro* and *in vivo* . Mol. Pharm. 22, 5346–5360. 10.1021/acs.molpharmaceut.5c00272 40717437

[B122] Zhao Y. Chen X. Huang Y. Zhang Z. Wang K. Zou D. (2024). Transcriptomic insights into hub genes, immune infiltration, and candidate drugs in erosive esophagitis. J. Inflamm. Res. 17, 7745–7760. 10.2147/JIR.S479032 39494202 PMC11529285

[B123] Zhao L. Lai Y. Jiao H. Li J. Lu K. Huang J. (2024). CRISPR-mediated Sox9 activation and RelA inhibition enhance cell therapy for osteoarthritis. Mol. Ther. 32, 2549–2562. 10.1016/j.ymthe.2024.06.016 38879753 PMC11405173

[B124] Zhong C. Xie T. Chen L. Zhong X. Li X. Cai X. (2022). Immune depletion of the methylated phenotype of colon cancer is closely related to resistance to immune checkpoint inhibitors. Front. Immunol. 13, 983636. 10.3389/fimmu.2022.983636 36159794 PMC9492852

[B125] Zhong H. Lu W. Tang Y. Wiel C. Wei Y. Cao J. (2023). SOX9 drives KRAS-induced lung adenocarcinoma progression and suppresses anti-tumor immunity. Oncogene 42, 2183–2194. 10.1038/s41388-023-02715-5 37258742 PMC11809655

[B126] Zhou T. Wu L. Ma N. Tang F. Yu Z. Jiang Z. (2020). SOX9-activated FARSA-AS1 predetermines cell growth, stemness, and metastasis in colorectal cancer through upregulating FARSA and SOX9. Cell Death Dis. 11, 1071. 10.1038/s41419-020-03273-4 33318478 PMC7736271

[B127] Zhou M. Lin B. Wu P. Ke Y. Huang S. Zhang F. (2024). SOX9 induces orbital fibroblast activation in thyroid eye disease *via* MAPK/ERK1/2 pathway. Invest Ophthalmol. Vis. Sci. 65, 25. 10.1167/iovs.65.2.25 38345552 PMC10866156

[B128] Zhu X. Huang H. Zong Y. Zhang L. (2022). SRY-related high-mobility group box 9 (SOX9) alleviates cigarette smoke extract (CSE)-induced inflammatory injury in human bronchial epithelial cells by suppressing stromal interaction molecule 1 (STIM1) expression. Inflamm. Res. 71, 565–576. 10.1007/s00011-022-01576-0 35488927

[B129] Zhuang X. Chen B. Huang S. Han J. Zhou G. Xu S. (2022). Hypermethylation of miR-145 promoter-mediated SOX9-CLDN8 pathway regulates intestinal mucosal barrier in Crohn's disease. EBioMedicine 76, 103846. 10.1016/j.ebiom.2022.103846 35124427 PMC8829091

